# Quantile regression analysis of the heterogeneous association between digital cultural engagement and health literacy: evidence from rural China

**DOI:** 10.3389/fpubh.2026.1802126

**Published:** 2026-04-09

**Authors:** Chenglin Du, Lanka Wu

**Affiliations:** 1 College of Foreign Languages and Literature, Chongqing University of Education, Chongqing, China; 2 School of Tourism and Hotel Management, University of Sanya, Sanya, China

**Keywords:** digital cultural engagement, digital divide, health equity, health literacy, intangible cultural heritage (ICH), quantile regression, rural China

## Abstract

**Background:**

The digital divide limits rural residents’ access to health information, and the potential of utilizing non healthy but culturally relevant digital resources to enhance health literacy is not yet clear.

**Methods:**

We conducted a cross-sectional survey of 1,457 adults across 32 traditional villages in Chongqing, China. Intangible cultural heritage digital-resource use and health literacy were assessed using validated scales. Associations were examined using quantile regression, adjusted for sociodemographic, digital-infrastructure and health-status covariates. Mediation analysis and E-value tests were performed to probe mechanisms and assess robustness.

**Results:**

Higher use of digital resources of intangible cultural heritage was significantly related to higher health literacy scores, and this correlation existed in all subgroups. However, the strength of this association increased progressively along the health literacy distribution: it was strongest at the 90th percentile (*β* = 2.49, 95% CI: 1.98–3.00) and weakest at the 10th percentile (*β* = 1.38, 95% CI: 0.95–1.81), supporting the cumulative advantage hypothesis. This association was more pronounced in low-bandwidth network villages (interaction *p* = 0.038 at the 10th percentile) and in the older population (≥ 60 years) (interaction *p* = 0.007 at the 10th percentile). Mediation analysis revealed that digital self-efficacy explained 31.7% of the total association between ICH use and health literacy, with significantly stronger mediation effects in older adults (45.2%) and low-bandwidth villages (38.5%). The E value for the main association (high frequency use of intangible cultural heritage reduces the risk of inadequate health literacy) was 3.24, indicating that the results are moderately robust to unmeasured confounders.

**Conclusion:**

The association between intangible cultural heritage digital resource use and health literacy exhibits a cumulative advantage pattern, strengthening progressively across the health literacy distribution. This association is particularly pronounced in low-bandwidth villages and among older adults, with digital self-efficacy mediating approximately one-third of the total effect. These findings highlight a culturally grounded pathway to promote health equity through targeted digital inclusion strategies.

## 1 Introduction

Health literacy, which refers to the ability to access, understand and apply health information to make informed decisions, is a key social factor affecting health and a cornerstone of reducing health disparities (1). In rural and digital-poor communities, lower health literacy exacerbates inequalities in access to health services, management of chronic diseases, and health outcomes (2). At the same time, the rapid digitization of health information and services has created a “digital health gap”, and those who lack digital access or skills face superimposed disadvantages when acquiring timely health knowledge (3). Strategies that have traditionally bridged this gap have typically focused on the direct delivery of health information through digital platforms (4). However, this approach largely ignores the key premise that basic digital literacy and self-efficacy need to be developed first, which is necessary for individuals to be able to effectively browse, evaluate, and utilize online information (5). This creates a fundamental paradox: interventions aimed at improving digital health literacy may be difficult to reach the people who need them most precisely because of the digital barriers people already have (6).

The digital preservation of intangible cultural heritage (ICH) provides a new and promising way for exploration (7). Intangible cultural heritage is an important carrier of community identity and social cohesion, and its digitization has become the strategic focus of sustainable cultural development (8). In a resource-constrained environment, the development of lightweight and culturally significant digital intangible cultural heritage resources provides a unique opportunity (9). Exposure to these familiar, low cognitive loads can serve as a context-specific digital literacy practice that may enhance overall digital comfort and skills and create a “spillover” path from cultural engagement to improved access to health information (10). This potential path merges with the cumulative advantage framework of “skills beget skills” and the concept of digital literacy as a basic and transferable ability to expand the latter (11).

A growing body of literature is exploring how engagement with non-health digital content can foster basic digital competence. For example, recent research on digital storytelling and community archiving has shown that culturally resonant materials can lower initial barriers to technology use, particularly among marginalized groups (12). However, much of this research remains focused on educational or social outcomes, and the specific mechanisms linking cultural digital resources to health-related competencies have not been systematically examined. In addition, the question of why digital intangible cultural heritage resources (as opposed to other forms of cultural content) may be particularly appropriate for rural residents remains underexplored. Intangible cultural heritage is deeply embedded in local traditions and daily life, which generates internal dynamics and shortens the psychological distance from digital engagement. Intangible cultural heritage materials are considered familiar and meaningful compared to general health information or entertainment content, thereby increasing the likelihood of ongoing interaction, especially among the older population and those with limited digital experience (13). This cultural proximity may transform the initial engagement with digital technology from a daunting task to an engaging cultural experience, making intangible cultural heritage a particularly effective “gateway” for building digital self-efficacy in rural settings.

However, this potential path involves an unresolved theoretical debate about the distribution of digital benefits. Mainstream digital divide models, such as the “access, skills, use” framework, effectively map differences in resource allocation, but provide limited insight into whether digital engagement is primarily reinforcing existing strengths or truly helping the most disadvantaged to achieve meaningful catch-up (14). Similarly, traditional health literacy interventions typically employ a “deficit model” that focuses on knowledge transfer and may ignore the role of non-cognitive factors such as motivation and self-efficacy, which are critical for skill acquisition and transfer (15). Thus, a key gap lies in understanding for whom and under what conditions culture-based digital engagement translates into health-related competencies (16). Methodological reliance on average-effect models, such as ordinary least-squares regression, further masks these underlying distribution dynamics by estimating a single average effect that may not accurately represent the relevance of the entire outcome distribution (17).

In response to these intertwined theoretical and empirical gaps, this study goes beyond the average effects model using a quantile regression framework to reveal a heterogeneous picture of the association between digital cultural engagement and health literacy. Specifically, we proposed and tested the following hypotheses: H1: The frequency of use of digital resources of intangible cultural heritage is positively related to health literacy scores, and this association is heterogeneous in the distribution of health literacy, which indicates that the strength of association for high quantiles (such as the 90th percentile) is greater than that for low quantiles (such as the 10th percentile). This is consistent with the cumulative advantage (or “skill begets skill”) model theorized in the sociology of educational and technological adoption. This pattern suggests that people with higher baseline health literacy have better information processing skills and learning strategies that allow them to derive greater benefits from any digital engagement, including culturally relevant content. H2: This positive relationship is moderated by environmental and personal factors, and is significantly stronger among residents of low-bandwidth network villages and among the older population (≥ 60 years old). The rationale for expecting stronger associations in low-bandwidth environments draws on the concept of “appropriate technology”: when infrastructure constraints restrict access to high-bandwidth applications, lightweight, culturally tailored content (such as ICH resources) becomes the primary and most meaningful digital experience, with a correspondingly greater impact on digital skills development. For older adults, socio-affective selectivity theory suggests that motivation shifts to emotionally meaningful goals with age; therefore, intangible cultural heritage content that resonates with individual and collective memory may elicit stronger engagement and greater self-efficacy, amplifying spillover effects on health literacy. H3: Digital self-efficacy plays a mediating role in the association between digital use of intangible cultural heritage and health literacy, and the strength of this mediating path is greater in the older population and low-bandwidth network villages. This hypothesis is based on social cognitive theory, which emphasizes mastery experience as a key source of self-efficacy. Successfully navigating familiar cultural content online can provide such mastery experiences, boosting digital confidence; this heightened sense of self-efficacy facilitates exploration of other online domains, including health information seeking. As stated by H2, mediation pathways are expected to be stronger in the subgroups where initial participation is most dynamic and influential, namely the older population and residents of low-bandwidth villages. This study explores not only whether there is an association between digital cultural participation and health literacy, but also under what conditions and through what potential mechanisms, who is most closely related to digital cultural participation, aiming to provide a detailed, equity-focused perspective to inform the formulation of comprehensive strategies. By integrating digital divide theory, cumulative advantage theory, and social cognitive theory, the study constructs a comprehensive theoretical framework linking cultural engagement to health literacy through the mediating role of digital self-efficacy and the moderating effects of age and infrastructure. Using cultural digitization to promote digital inclusion and health literacy in marginalized communities. Supplementary Figure 1 provides an overview of the analytical framework, describing the hypothesized relationships between ICH use, digital self-efficacy, health literacy, and the moderating role of age and bandwidth.

## 2 Methods

### 2.1 Study design and participants

This study was a population-based cross-sectional survey conducted in Chongqing, China, from May to August 2024. The target population is adult residents (age ≥ 18 years) living in officially recognized traditional villages known for their well-preserved buildings and cultural heritage. Inclusion criteria were: (1) residence in the current village for at least 6 months; (2) basic understanding and presentation skills to complete the questionnaire; and (3) provision of written informed consent for voluntary participation. Individuals who were unable to complete the questionnaire due to severe cognitive or communication impairments were excluded. We used a multi-stage stratified cluster sampling design. According to two criteria, four districts and counties with dense traditional villages were deliberately selected: (a) the most concentrated traditional villages officially recognized by Chongqing, and (B) the diversity representing the level of economic development (low, medium and high per capita GDP) to improve the representativeness of the sample. Eight townships were randomly selected from these areas. Thirty-two natural villages were randomly selected from the selected townships and stratified according to the quality of the local broadband infrastructure (low: ≤ 10 Mbps vs. medium/high: > 10 Mbps). Within each selected village, approximately 45–50 households were recruited using a systematic random sampling method, and one eligible adult from each household was invited to participate. Individuals who were unable to complete the questionnaire due to severe cognitive or communication impairment were excluded.

### 2.2 Sample size calculation

The sample size calculation aims to test the association between the utilization frequency of intangible cultural heritage and health literacy scores. Based on a pre-survey (*n =* 120), the expected Spearman correlation coefficient is 0.15. To detect this association with a statistical power of 90% at a two-sided alpha level of 0.05, a minimum of 862 participants are required (18). The target sample size was set at 1,520, taking into account the 1.5 design effect of cluster sampling and the expected 85% response rate. The final sample of 1,457 individuals met this requirement. To address the need for quantile regression to estimate extreme quantiles, we also refer to the methodological literature, which States that in social science research, a sample size greater than 1,000 is usually sufficient for stable estimation of multiple quantiles (19). In addition, for the planned mediation analysis, a Monte Carlo simulation (20,000 replicates) showed that this sample provided more than 85% statistical power to detect an indirect effect of 15% of the total effect, assuming a correlation coefficient of 0.25 between the mediating variable and the outcome variable (20).

### 2.3 Measurement and data collection

Trained researchers conducted face-to-face interviews using a structured electronic questionnaire on a tablet computer.

#### 2.3.1 Primary exposure: ICH digital resource utilization frequency

Frequency of use was measured by a 10-item scale developed for this study based on the Technology Acceptance Model (21, 22). The scale was developed through the following steps: (1) qualitative interviews with residents and ICH practitioners to generate entries; (2) expert review by sociologists and digital media scholars to ensure content validity; and (3) pretesting (*n =* 50) to ensure clarity of entries. The pretest sample included 50 residents from two traditional villages not included in the main survey. Item analysis revealed that all items had a corrected total item correlation of more than 0.40, and no items were removed due to ceiling or floor effects. Exploratory factor analysis (principal axis factorization) indicated that the single factor structure (eigenvalue = 4.82) explained 48.2% of the total variance, with factor loadings ranging from 0.51 to 0.79, supporting the unidimensionality of the scale. The scale uses a 5-point Likert scale (1 = never, 5 = daily) to assess the frequency of using locally-developed, low-bandwidth environments adapted intangible cultural heritage resources. The total score was the average of all entries (range 1–5). The scale showed good internal consistency reliability in this sample (Cronbach’s *α* = 0.87). To further determine construct validity, confirmatory factor analysis was conducted. One-factor models showed an acceptable fit, supporting the unidimensionality of the scale (CFI = 0.95, TLI = 0.94, RMSEA = 0.052, SRMR = 0.043).

#### 2.3.2 Primary outcome: health literacy

Health literacy was assessed using the validated 53-item Health Literacy Questionnaire for Chinese Citizens (23). The questionnaire measures three dimensions: health knowledge and beliefs (17 items), healthy lifestyles and behaviors (20 items), and health skills (16 items). The total score (range 0–100) was calculated according to the standard algorithm. In the current rural sample, the scale demonstrated good reliability (Cronbach’s alpha = 0.91 for the total scale; range 0.78 to 0.86 for the subscales). Confirmatory factor analysis confirmed a three-factor structure (CFI = 0.92, TLI = 0.91, RMSEA = 0.058), indicating acceptable construct validity for this population. In predictive modeling, the categorical outcome of “inadequate health literacy” was defined as a total score of 26 points below the national threshold.

#### 2.3.3 Covariates and other measures

Data were collected on potential confounders and effect modifiers, including: Socio-demographic characteristics: age (continuous variable), gender, education level (classified as primary school and below, junior high school, senior high school and above), annual household income. Digital access and Context: village bandwidth levels (verified by local service providers: low/≤ 10 Mbps, medium/11–50 Mbps, high/> 50 Mbps), personal smartphone ownership (yes/no), and a 5-item digital self-efficacy scale adapted from existing instruments (*α* = 0.79) (24). Health status: Self-rated health status (5-point scale, dichotomized as poor/fair vs. good/very good), number of chronic diseases diagnosed by physicians. The selection of these covariates was based on previous literature on health literacy and digital divide research (2, 5, 14), which identified sociodemographic factors, digital access, and health status as key determinants of digital participation and health literacy outcomes.

### 2.4 Statistical analysis

Descriptive statistics were used to summarize participant characteristics by health literacy quintiles, and differences between groups were assessed by analysis of variance, Kruskal-Wallis test, or chi-square test as appropriate. The primary analysis used quantile regression to simulate the association between the frequency of intangible cultural heritage use (a continuous predictor variable) and health literacy scores at the 10, 25, 50, 75, and 90% quantiles of the outcome distribution. These quantiles were selected following standard practice in health disparities research (25) to capture both ends of the distribution (Q10, Q90) where equity concerns are most salient, as well as central tendency (Q50). This approach allows for a comprehensive assessment of heterogeneity across the full spectrum of health literacy. We estimate a range of models, starting with rough associations and adjusting for demographics, socioeconomic status, digital infrastructure, and health status. The regression coefficients and their 95% confidence intervals were derived using Bootstrap replicates (1,000 replicates) for robust standard errors, and a village-level cluster sampling design was considered. The Wald test was used to formally test for heterogeneity in the strength of association between different quantiles. Subsequent analyses investigated potential effect modifiers by performing hierarchical quantile regressions on village bandwidth levels and age groups, respectively, and formal interaction tests using likelihood ratio tests. To explore the mechanisms underlying the observed associations, we conducted a causal mediation analysis using the coefficient product method within a counterfactual framework, implemented using the “mediation” package in R. The model specified ICH use as the exposure variable, digital self-efficacy scores as the mediator variable, and health literacy scores as the outcome variable, adjusting for all covariates. Standard errors and confidence intervals for indirect effects were estimated using nonparametric bootstrap with 1,000 resamples. We further performed an E-value analysis to quantify how strongly an unmeasured confounder would need to be associated with both the exposure variable and the outcome variable to fully explain the observed association between high frequency of intangible cultural heritage use and reduced risk of inadequate health literacy. The E-value was calculated using the formula: E-value = RR + sqrt(RR × (RR − 1)) for risk ratios, higher E-values indicate greater robustness to unmeasured confounding. In addition, multivariate logistic regression was used to assess the predictive value of intangible cultural heritage participation for identifying health literacy deficiencies. Model performance was assessed by the area under the receiver operating characteristic curve, sensitivity, specificity, and goodness-of-fit tests. To assess potential multicollinearity among predictors, variance inflation factors (VIF) were calculated for all regression models; all VIF values were below 3, indicating no serious multicollinearity concerns. All statistical analyses were performed using R software (version 4.4.2). A two-sided *p* < 0.05 was considered statistically significant. Given the cross-sectional design, we acknowledge the possibility of reverse causation (for example, people with higher health literacy may be more likely to have access to digital ICH resources). While the current design is unable to definitively determine the causal direction, the E-value analysis provides some indication of robustness to unmeasured confounding, and the theoretical framework (Skill Produces Skill) suggests a two-way reinforcement process worthy of future longitudinal investigation.

## 3 Results

### 3.1 Characteristics of the study population across health literacy quintiles

A total of 1,457 rural residents were included in the final analysis. To clarify the underlying social gradient, participants were divided into five equal groups (Q1 to Q5) based on the total health literacy score, with Q1 representing the lowest 20% and Q5 representing the highest 20%. The baseline characteristics of each subgroup are shown in Table 1. Obvious socioeconomic and numerical gradients are evident. Compared with the highest subgroup (Q5), participants in the lowest subgroup of health literacy (Q1) were significantly older (mean age 61.8 years, SD 12.9), had significantly less education (62.8% had a primary education or less), and reported a lower median annual household income (3.2 × 10^4^CNY, interquartile range 2.1–4.6). A significant digital divide was also observed: 68.1% of participants in the Q1 group lived in low-bandwidth villages (≤ 10 Mbps), compared to only 28.8% in the Q5 group. Critically, the main exposure variable, the frequency of use of digital intangible cultural heritage resources, showed a strong, monotonically increasing gradient, rising from a mean of 1.8 (standard deviation 0.8) in the Q1 group to 3.7 (standard deviation 0.9) in the Q5 group (trend *p* value < 0.001, linear contrast test). Similar gradient changes were observed in smartphone ownership, digital self-efficacy, and health status indicators, confirming the multifaceted nature of health literacy differences in this population. Preliminary bivariate correlation analysis showed that ICH use frequency was positively associated with health literacy scores (Pearson R = 0.45, 95% CI: 0.41–0.49, *p* < 0.001). Digital self-efficacy was also significantly associated with ICH use (R = 0.38, 95% CI: 0.34–0.42, *p* < 0.001) and health literacy (R = 0.52, 95% CI: 4.48–0.56, *p* < 0.001), providing initial support for the proposed mediation pathway. In addition, there was a modest but significant correlation between village bandwidth levels and ICH use (point-to-point R = 0.21, *p* < 0.001), indicating that better infrastructure is associated with higher participation, but the correlation was not conclusive. All values in Table 1 are presented in a consistent format, with means and standard deviations to two decimal places and percentages reported without decimal points for clarity.

**Table 1:** Baseline characteristics of study participants by health literacy quintiles (*N =* 1,457).

Characteristic	Overall (*N =* 1,457)	Q1 (Lowest) (*n =* 298)	Q2 (*n =* 291)	Q3 (*n =* 289)	Q4 (*n =* 291)	Q5 (Highest) (*n =* 288)	P for Trend
Demographics							
Age, mean (SD), years	53.2(14.7)	61.8 (12.9)	56.1 (13.6)	51.4 (13.2)	48.7 (12.4)	45.9 (11.8)	<0.001
Female, *n* (%)	773 (53.1)	164 (55.0)	158 (54.3)	151 (52.2)	151 (51.9)	149 (51.7)	0.214
Socioeconomic status							
Education level, *n* (%)							
Primary school or below	431 (29.6)	187 (62.8)	132 (45.4)	83 (28.7)	54 (18.6)	30 (10.4)	<0.001
Middle school	624 (42.8)	94 (31.5)	126 (43.3)	151 (52.2)	151 (51.9)	102 (35.4)	
High school or above	402 (27.6)	17 (5.7)	33 (11.3)	55 (19.0)	86 (29.6)	156 (54.2)	
Annual household income, median (IQR), 10^4^ CNY	4.3 (2.9–6.2)	3.2 (2.1–4.6)	3.9 (2.6–5.6)	4.4 (3.1–6.3)	5.1 (3.6–7.2)	5.9 (4.2–8.1)	<0.001
Digital environment and access							
Village bandwidth level: Low (≤10 Mbps), *n* (%)	732 (50.2)	203 (68.1)	166 (57.0)	141 (48.8)	112 (38.5)	83 (28.8)	<0.001
Personal smartphone ownership, *n* (%)	1,238 (85.0)	217 (72.8)	241 (82.8)	256 (88.6)	265 (91.1)	259 (89.9)	<0.001
Digital self-efficacy score, mean (SD)	2.8 (0.9)	2.0 (0.7)	2.5 (0.7)	2.9 (0.7)	3.2 (0.7)	3.6 (0.6)	<0.001
Health status and behavior							
Self-rated health: Poor/Fair, *n* (%)	583 (40.0)	190 (63.8)	148 (50.9)	111 (38.4)	82 (28.2)	52 (18.1)	<0.001
No. of chronic conditions, mean (SD)	1.2 (1.1)	1.7 (1.2)	1.3 (1.1)	1.1 (1.0)	0.9 (0.9)	0.7 (0.8)	<0.001
Primary study variable							
ICH utilization frequency, mean (SD)	2.7 (1.2)	1.8 (0.8)	2.3 (0.9)	2.7 (0.9)	3.2 (1.0)	3.7 (0.9)	<0.001

### 3.2 Quantile specific associations between ICH digital engagement and health literacy

**Figure 1:**
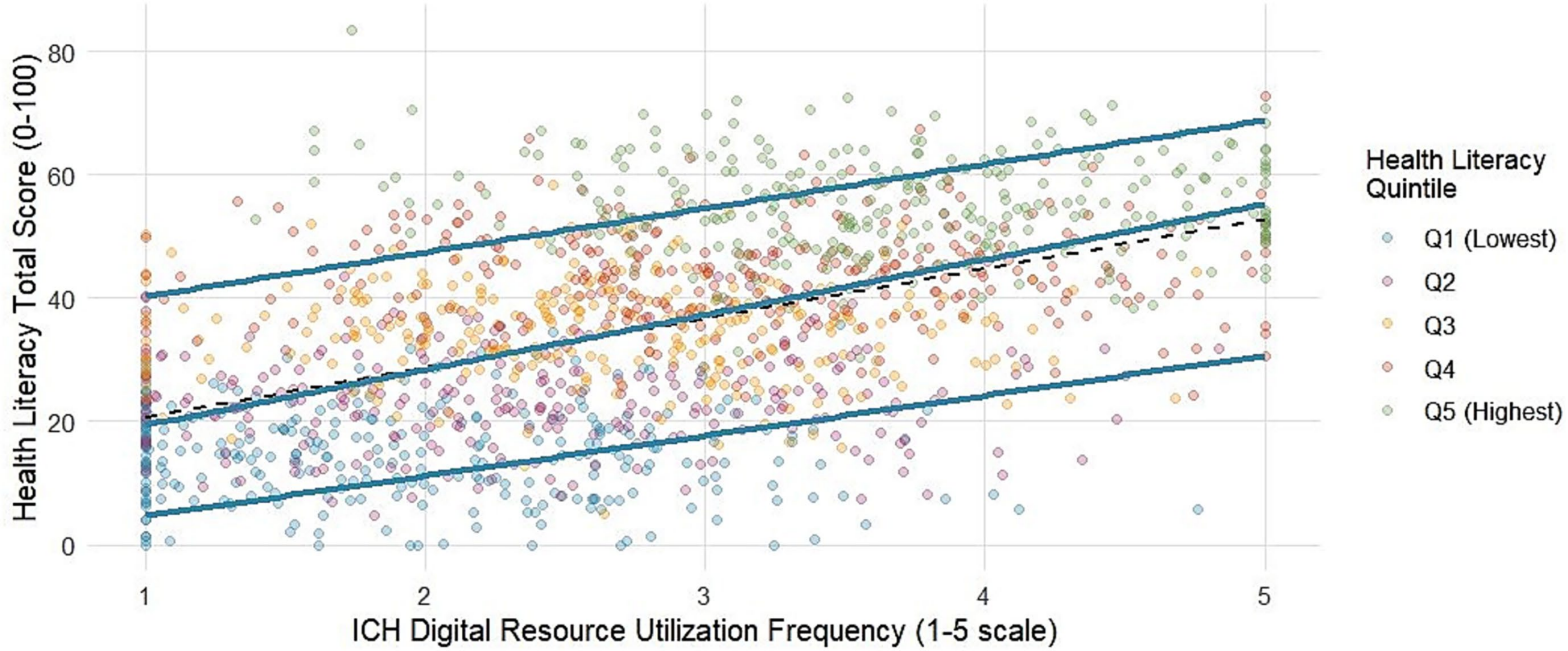
Bivariate association between ICH digital use and health literacy with regression lines. This scatter density plot demonstrates the relationship between the frequency of use of digital resources of intangible cultural heritage (1–5 subscales) and the total score of health literacy (0–100 points) among rural residents (*N =* 1,457). The density of the points is represented by a background color gradient (warm colors represent higher density). The red dashed line represents the ordinary least squares regression fit line (average effect). The blue solid lines represent the quantile regression fit lines for the 10, 50% (median), and 90% quantiles of the health literacy distribution, visually demonstrating the heterogeneity in the strength of the association.

**Figure 2:**
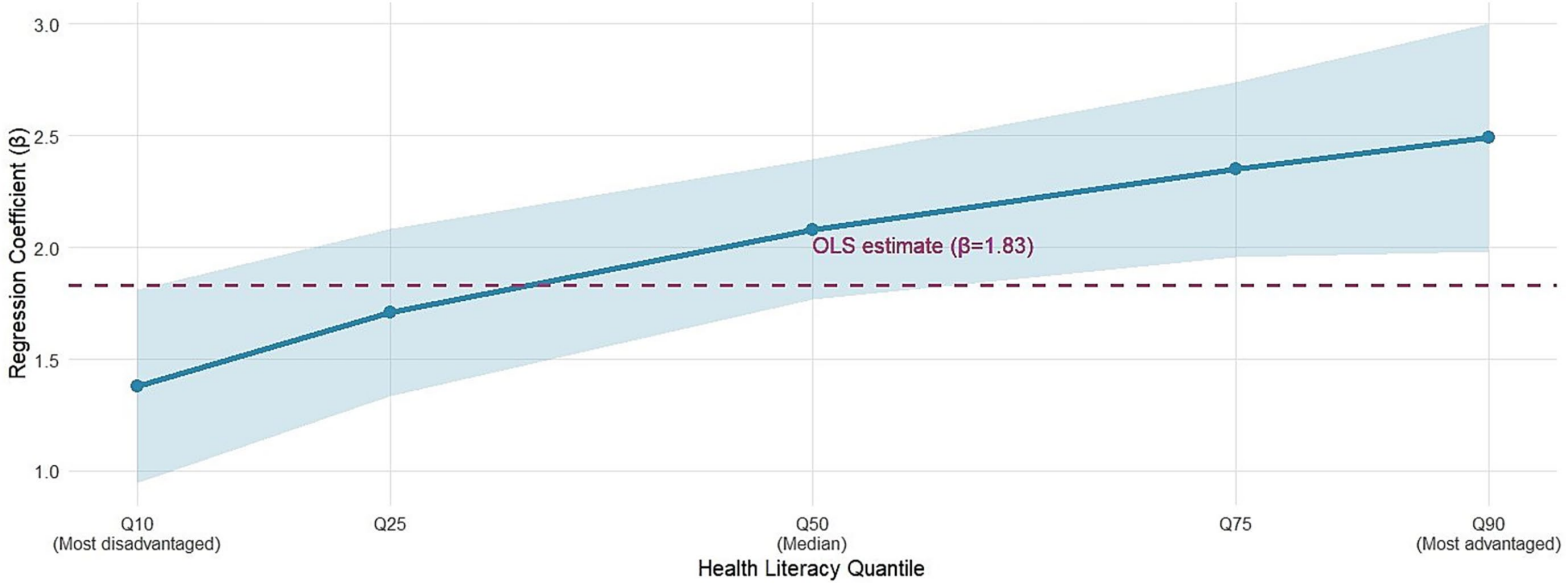
Trajectory of quantile regression coefficients for ICH use on health literacy. The figure, based on the fully adjusted model, shows the regression coefficient (*β*) for the use of intangible cultural heritage digital resources (each additional point) on the conditional quantile of the health literacy distribution (from 0.10 to 0.90). The solid blue line and shaded band represent the point estimate and 95% confidence interval, respectively. The red horizontal dashed line indicates the ordinary least squares regression coefficients used for comparison. The upward trajectory indicates that the positive association between ICH use and health literacy is significantly stronger for individuals in the higher quantiles of the health literacy distribution.

The bivariate relationship between the use frequency of intangible cultural heritage and the health literacy score is visually presented in Figure 1. The scatter density plot is superimposed with the ordinary least squares regression line and the quantile regression line. The graph shows a positive correlation and considerable heterogeneity in the data distribution, which justifies the use of quantile regression. The core results of quantile regression analysis are shown in Table 2. After fully adjusting for age, gender, education, income, bandwidth level, and chronic disease status, a 1-point increase in the frequency of ICH use was associated with a statistically significant increase in health literacy scores across all quantiles examined. However, the strength of the association is not uniform. The association was strongest at the upper end of the health literacy distribution (90th quantile: *β* = 2.49, 95% confidence interval: 1.98, 3.00) and weakest at the lower end (10th quantile: *β* = 1.38, 95% confidence interval: 0.95, 1.81). A formal Wald test confirmed that the coefficients were significantly heterogeneous in distribution (*p* = 0.003 for Q10 equal to Q90). The ordinary least squares regression estimate (*β* = 1.83) represents an average effect that masks the underlying pattern of increasing association for individuals with higher levels of health literacy. The OLS coefficient lies between estimates of Q10 and Q90, suggesting that a mean-based approach would underestimate the potential benefit for the healthiest population (36%, or 2.49 vs. 1.83) and overestimate the benefit for the unhealthiest population (33%, or 1.38 vs. 1.83). This disparity highlights the importance of studying distributional effects: interventions that target digital resources of intangible cultural heritage may inadvertently widen inequalities in health literacy if they disproportionately benefit those already at the top. Stronger associations at higher quantiles support the cumulative advantage hypothesis (H1), suggesting that individuals with higher baseline health literacy are better able to translate cultural digital engagement into further health literacy gains. This heterogeneity is captured in the coefficient trace plot of Figure 2. The drawn curve shows a steady upward trend, indicating that the positive association of digital participation in intangible cultural heritage gradually becomes stronger for individuals with higher levels of health literacy. The ordinary least squares regression estimates, shown as horizontal dashed lines, lie below the quantile regression curve over most of the interval of the distribution, especially at the upper quantiles. The 95% confidence in Figure 2 brings self-lifting sampling, reflecting the precision of the quantile estimates; the rising slope remains clearly discernible across the distribution, enhancing the robustness of the heterogeneity pattern.

**Table 2:** Quantile regression analyses of the association between ICH digital resource utilization frequency and health literacy total score.

Model/quantile	*β* coefficient	Robust SE	95% CI	*P-*value	OLS *β* (for comparison)
Q10 (Most Disadvantaged)	1.38	0.22	(0.95, 1.81)	<0.001	1.83
Q25	1.71	0.19	(1.34, 2.08)	<0.001	
Q50 (Median)	2.08	0.16	(1.77, 2.39)	<0.001	
Q75	2.35	0.20	(1.96, 2.74)	<0.001	
Q90 (Most Advantaged)	2.49	0.26	(1.98, 3.00)	<0.001	
Test of Equality (Q10 vs. Q90)	P = 0.003				
Test of Equality (Q25 vs. Q75)	*p* = 0.021				

All models are fully adjusted for age, sex, education, household income, village bandwidth level, and number of chronic conditions. SE, Standard Error; CI, Confidence Interval; OLS, Ordinary Least Squares (linear regression) coefficient from a fully adjusted model.

### 3.3 Stratified analyses: effect modification by infrastructure and age

**Figure 3:**
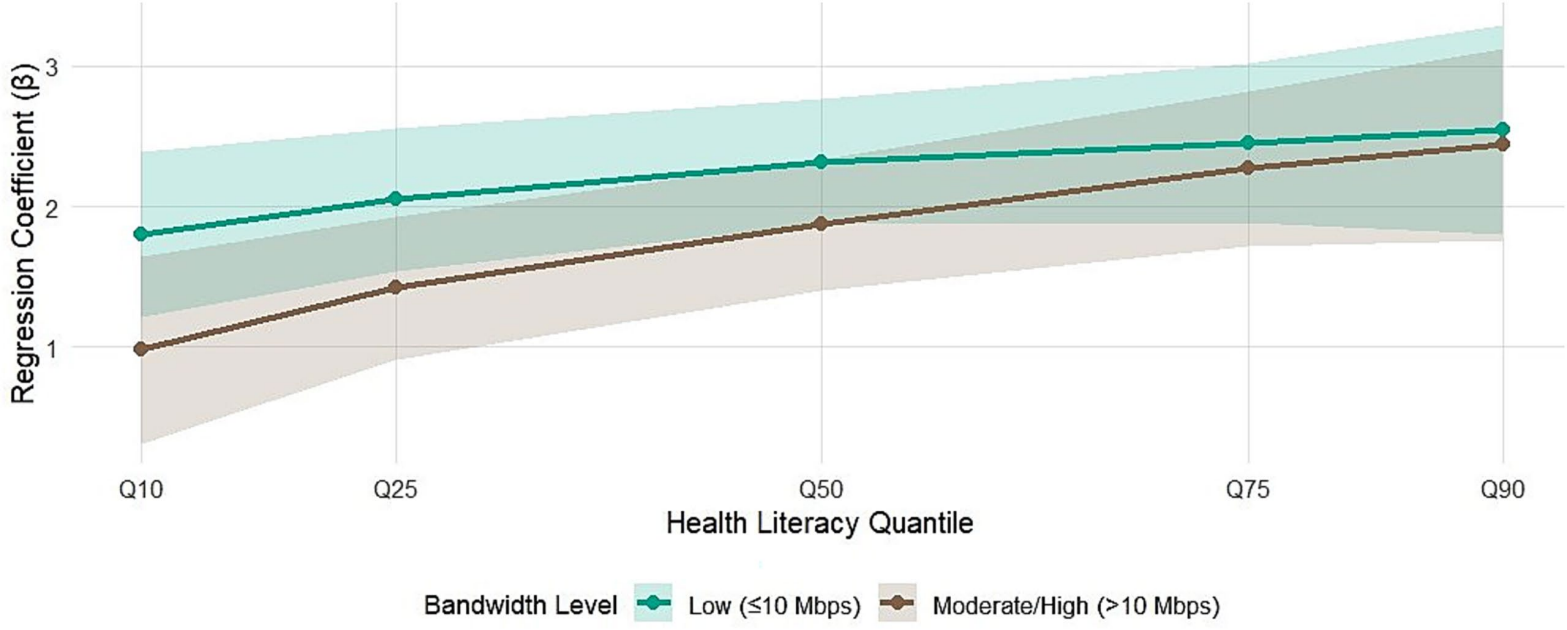
Quantile regression coefficients stratified by village bandwidth level. Compare the association between ICH use and health literacy at different quantiles in low bandwidth villages (≤ 10 Mbps, orange line) and medium/high bandwidth villages (> 10 Mbps, purple line). Error bars represent 95% confidence intervals. The stronger coefficients for low-bandwidth villages across most quantiles (especially the low-end 10 th, 25th) suggest that the impact of this association is greater in infrastructure-constrained environments.

**Figure 4:**
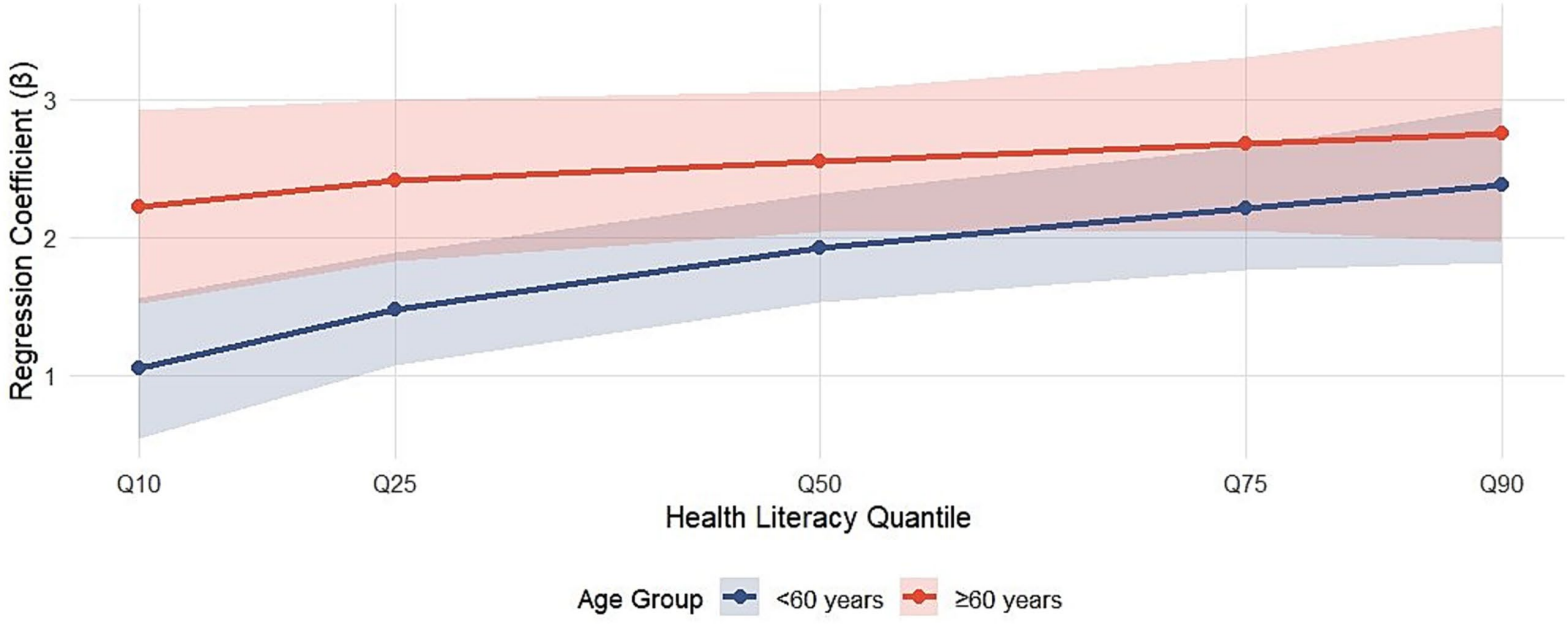
Quantile regression coefficients are stratified by age group. To compare the association between intangible cultural heritage use and health literacy at different quantiles in older adults (≥ 60 years, golden line) and younger adults (< 60 years, turquoise line). Error bars represent 95% confidence intervals. Persistent and significantly stronger coefficients across all quantiles for the older adult group highlight that this population is a key beneficiary of digital cultural engagement.

The association between ICH use and health literacy was significantly modified by village digital infrastructure and participant age. The detailed results of the hierarchical quantile regression are presented in Tables 3, 4 and are visually synthesized in Figures 3, 4. At almost all quantiles, the association strength of low-bandwidth villages is persistent and significantly stronger. This difference is most pronounced at the lower end of the health literacy distribution. For example, among the most disadvantaged individuals (10th quantile), the strength of the association was almost twice as strong for low-bandwidth villages (*β* = 1.80, 95% confidence interval: 1.20, 2.40) as for medium/high-bandwidth villages (*β* = 0.98, 95% confidence interval: 0.31, 1.65), with a statistically significant interaction test (*p* = 0.038). Figure 3 provides a powerful visual representation of these numerical results. The coefficient trace plot clearly shows that the orange line (low bandwidth village) is above the purple line (medium/high bandwidth village) throughout the distribution. The visual gap between the two lines is widest at the lower quantiles (Q10, Q25), visually demonstrating the location where the infrastructure effect modification is strongest, which coincides perfectly with the interaction term that is significant at the 10th quantile. This pattern suggests that in resource-constrained environments, ICH digital resources may serve as unique and valuable gateways for digital skills development, which is consistent with the “appropriate technology” hypothesis. The fact that this gap narrows at higher quantiles implies that once basic digital capabilities are achieved, the limitations of infrastructure are less important for leveraging cultural content. A significant and consistent modification pattern of age effects was observed. The data in Table 4 show that older adults (≥ 60 years) show significantly stronger associations across each examined quantile. For older adults with very low health literacy (Q10), a 1-point increase in ICH use was associated with a 2.22-point increase in health literacy score (95% confidence interval: 1.51, 2.93), which is the strength of the association observed in young adults in the same quantile (*β* = 1.05, 95% confidence interval: 0.54, 1.56) (interaction *p*-value = 0.007). Figure 4 effectively captures this life-stage gradient. The plotted coefficient trajectories show two clearly separated, nearly parallel lines, with the golden line (older adults) lying above the turquoise line (younger adults) across the health literacy spectrum. This visual presentation highlights the general and powerful role of age in moderating the strength of the association between intangible cultural heritage and health literacy, reinforcing the statistical evidence of the interaction. The persistent strengths of older adults are consistent with social–emotional selectivity theory: culturally familiar content resonates more deeply with older adults, resulting in greater intrinsic motivation and engagement, which in turn amplifies spillover effects on health literacy.

**Table 3:** Stratified quantile regression analysis by village bandwidth level.

Quantile	Low-bandwidth villages (≤10 Mbps, *n =* 732)		Moderate/high-bandwidth villages (>10 Mbps, *n =* 725)		*P* for interaction
	*β* (95% CI)	*P-*value	*β* (95% CI)	*P*-value	
Q10	1.80 (1.20, 2.40)	<0.001	0.98 (0.31, 1.65)	0.004	0.038
Q25	2.05 (1.54, 2.56)	<0.001	1.42 (0.90, 1.94)	<0.001	0.079
Q50 (Median)	2.32 (1.87, 2.77)	<0.001	1.87 (1.40, 2.34)	<0.001	0.162
Q75	2.45 (1.89, 3.01)	<0.001	2.27 (1.72, 2.82)	<0.001	0.595
Q90	2.55 (1.80, 3.30)	<0.001	2.44 (1.75, 3.13)	<0.001	0.812

All models are adjusted for age, sex, education, household income, and number of chronic conditions. CI = Confidence Interval. P for interaction derived from a likelihood ratio test comparing models with and without a cross-product term between ICH utilization frequency (continuous) and the village bandwidth stratum. A significant *P-*value indicates that the association between ICH use and health literacy differs statistically between the two bandwidth groups at that quantile. The relative strength of the association within each stratum can be compared directly by examining the coefficient (*β*) values.

**Table 4:** Stratified quantile regression analysis by age group.

Quantile	Older adults (≥60 years, *n =* 478)		Younger adults (<60 years, *n =* 979)		*P* for interaction
	*β* (95% CI)	*P*-value	*β* (95% CI)	*P*-value	
Q10	2.22 (1.51, 2.93)	<0.001	1.05 (0.54, 1.56)	<0.001	0.007
Q25	2.41 (1.82, 3.00)	<0.001	1.48 (1.07, 1.89)	<0.001	0.010
Q50 (Median)	2.55 (2.04, 3.06)	<0.001	1.92 (1.53, 2.31)	<0.001	0.030
Q75	2.68 (2.05, 3.31)	<0.001	2.21 (1.75, 2.67)	<0.001	0.185
Q90	2.75 (1.96, 3.54)	<0.001	2.38 (1.81, 2.95)	<0.001	0.401

All models are adjusted for sex, education, household income, village bandwidth level, and number of chronic conditions. CI, Confidence Interval. P for interaction derived from a likelihood ratio test comparing models with and without a cross-product term between ICH utilization frequency (continuous) and the age group stratum. A significant *P* value indicates that the association between ICH use and health literacy differs statistically between the two age groups at that quantile. The relative strength of the association within each stratum can be compared directly by examining the coefficient (*β*) values.

### 3.4 Mediation analysis: the role of digital self efficacy

To explore the underlying mechanisms behind the primary associations, we investigated the role of digital self-efficacy as a mediating variable. Mediation analysis after adjustment for all covariates showed a significant indirect effect (*β* = 0.58, 95% confidence interval: 0.42, 0.75; *p* < 0.001). This suggests that approximately 31.7% (*β* _ total = 1.83) of the total association between ICH use and health literacy is mediated by increased digital self-efficacy. The proportion of mediation in the older population subgroup (45.2%) was significantly higher than that in the young adult subgroup (22.1%, difference *p* value = 0.012), and the proportion in the low-bandwidth villages (38.5%) was significantly higher than that in the medium/high-bandwidth villages (25.3%, difference *p* = 0.034). The greater mediating effect among older adults (45.2% vs. 22.1%) can be explained through the lens of social cognitive theory: for this group, successfully browsing culturally familiar digital content provides a particularly powerful mastery experience because the content itself has emotional and identity-related meanings. This heightened emotional engagement may translate into a greater boost in digital confidence, thereby facilitating health information seeking. Similarly, in low-bandwidth settings where digital options are limited, focused use of lightweight ICH resources may lead to more focused gains in self-efficacy, explaining a higher proportion of mediators (38.5% vs. 25.3%). These findings provide initial empirical support for the proposed culture-driven capacity spillover model, which argues that cultural engagement builds digital confidence and, in turn, facilitates access to health information. The complete results of the mediation analysis are shown in Table 5.

**Table 5:** Mediation analysis of the association between ICH digital resource use and health literacy: the role of digital self-efficacy.

Sample/subgroup	Total effect (c)	Direct effect (c’)	Indirect effect (ab)	Proportion mediated
Full Sample (*n =* 1,457)	1.83 (1.50, 2.16)	1.25 (0.92, 1.58)	0.58 (0.42, 0.75)	31.7%
By age group				
≥60 years (*n =* 478)	2.55 (2.04, 3.06)	1.40 (0.95, 1.85)	1.15 (0.85, 1.48)	45.2%
<60 years (*n =* 979)	1.92 (1.53, 2.31)	1.50 (1.12, 1.88)	0.42 (0.25, 0.61)	22.1%
P for difference	<0.001	0.365	0.012	
By bandwidth level				
Low-Bandwidth Villages (*n =* 732)	2.32 (1.87, 2.77)	1.43 (0.98, 1.88)	0.89 (0.62, 1.18)	38.5%
Moderate/High-Bandwidth Villages (*n =* 725)	1.87 (1.40, 2.34)	1.40 (0.95, 1.85)	0.47 (0.28, 0.68)	25.3%
P for difference	0.045	0.812	0.034	

All models are adjusted for age, sex, education, household income, village bandwidth, and number of chronic conditions. The Total Effect (c) is the association between ICH use and health literacy. The Direct Effect (c’) is this association after controlling for the mediator (digital self-efficacy). The Indirect Effect (ab) is the component mediated through digital self-efficacy. All values are standardized *β* coefficients with 95% confidence intervals in parentheses, derived from bias-corrected bootstrap analysis (1,000 replications). The P for difference refers to the significance test for between-subgroup differences in the corresponding effect estimate.

### 3.5 Predictive utility and sensitivity analysis for inadequate health literacy

**Figure 5:**
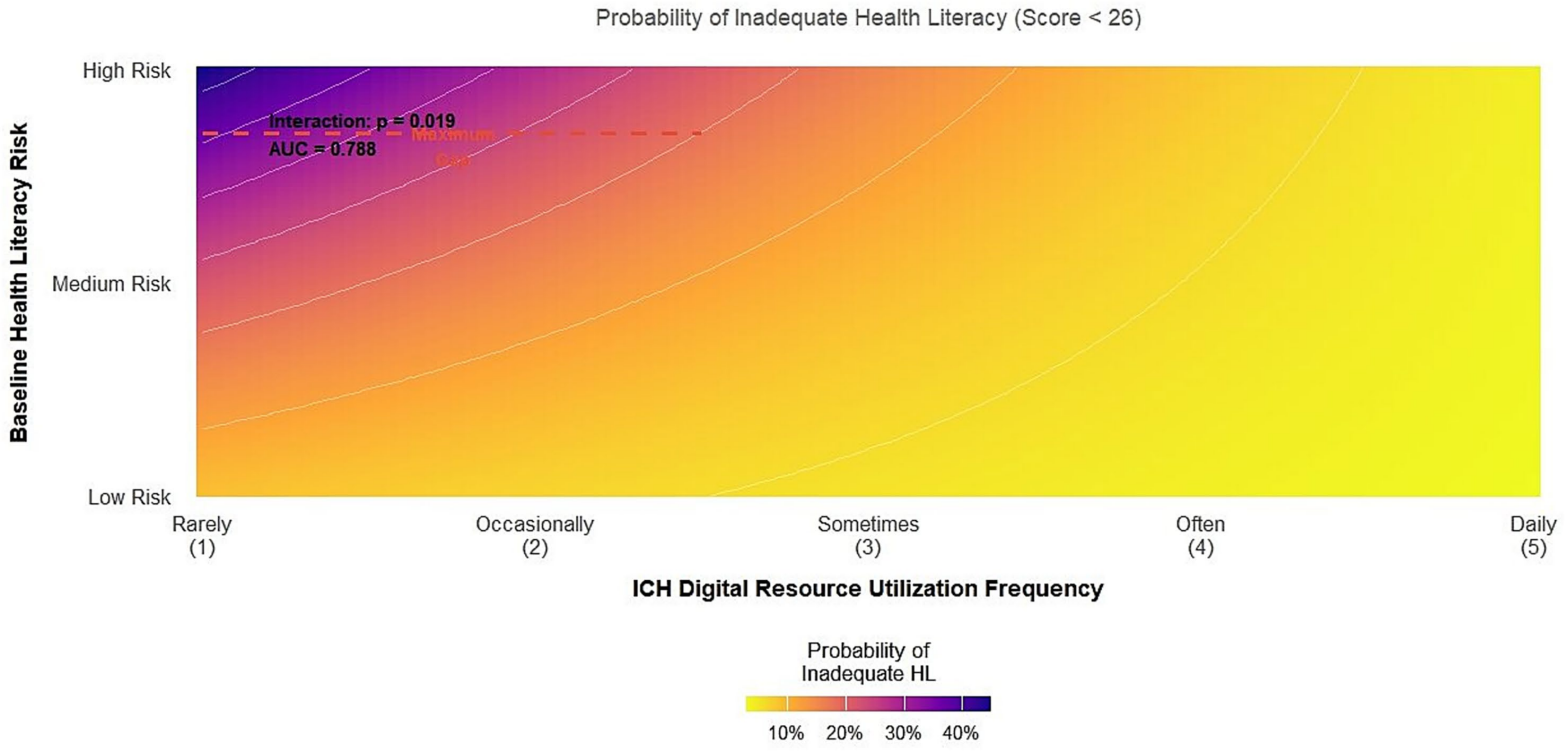
Interaction between ICH use and baseline risk on the predicted probability of inadequate health literacy. This three-dimensional thermal map visualizes the results of the logistic regression model. The *x*-axis represents the frequency of use of digital intangible cultural heritage resources, the *y*-axis represents the baseline health literacy risk profile (a continuous composite score), and the color gradient represents the predicted probability of having a health literacy deficiency (score < 26). Contour lines are superimposed in the figure. The “largest intervention gap” (dashed red box) marks the high-risk, low-engagement area where targeted interventions are most needed. The “optimal benefit zone” (green arrow) marks the area where high participation can significantly reduce the risk probability of high-risk individuals, indicating a significant interaction effect.

We assessed whether the use of intangible cultural heritage can enhance the identification of individuals with insufficient health literacy (score < 26). A logistic regression model including the interaction between the utilization level of intangible cultural heritage and the baseline risk stratification was established (Table 6). The model showed good performance with an area under the curve of 0.788 (95% confidence interval: 0.760, 0.816). High-frequency use of ICH resources was associated with a 61% reduction in the odds of inadequate health literacy (adjusted odds ratio = 0.39, 95% confidence interval: 0.27, 0.57). A significant interaction term (adjusted odds ratio = 0.52, *p* = 0.019) indicated that the negative association between high-frequency ICH use and inadequate health literacy was particularly strong for individuals initially classified as high-risk. To assess the robustness of this key finding to potential unmeasured confounders, we calculated the E value. The adjusted odds ratio for the observed high use of intangible cultural heritage was 0.39. The E value was 3.24, meaning that an unmeasured confounder would need to be at least 3.24 times more likely to be associated with both a high intangible cultural heritage use and a lower risk of inadequate health literacy (over and above the measured covariates) to fully explain the association. The lower limit of the 95% confidence interval (0.27) corresponds to an E value of 2.47. These values indicate that while the association is not completely immune to confounding effects, a moderately strong and unaccounted confounding factor is required to invalidate it. Figure 5 visualizes this interaction as a three-dimensional heat map. In this figure, the x-axis represents the frequency of use of digital resources of intangible cultural heritage, the y-axis represents the baseline health literacy risk status (continuous composite score), and the color gradient (from blue to red) represents the predicted probability of insufficient health literacy. The superimposed contour lines help to identify risk areas. The red dashed box highlights the “largest intervention gap,” an area where individuals are at high baseline risk but have low ICH engagement, indicating a priority target for intervention. The green arrow points to the “best benefit zone” where high ICH engagement greatly reduces the predicted risk for high-risk individuals, illustrating a significant interaction effect. All graphic elements (axis labels, legends, and contour lines) have been enlarged and clarified to ensure readability, and graphic captions now provide detailed descriptions of each component, making the graphic self-sufficient. The thermodynamic plot clearly shows that the greatest decrease in predicted risk occurs when high-risk populations increase their participation in ICH.

**Table 6:** Logistic regression predicting inadequate health literacy (Score <26) with ICH utilization and baseline risk stratum interaction.

Predictor	Adjusted odds ratio (aOR)	95% CI	*P-*value	Contribution to fit (%) #
Age (per 5-year increase)	1.31	(1.20, 1.43)	<0.001	14.8
Sex: Female (Ref: Male)	0.96	(0.77, 1.20)	0.735	0.1
Education (Ref: ≤Primary)				
Middle school	0.51	(0.39, 0.67)	<0.001	12.5
High school or above	0.27	(0.19, 0.38)	<0.001	23.9
ICH Utilization (Ref: Low)				
Moderate	0.63	(0.48, 0.82)	<0.001	7.9
High	0.39	(0.27, 0.57)	<0.001	13.1
Baseline HL Risk: High (Ref: Low)^**‡**^	3.85	(2.95, 5.02)	<0.001	18.2
Interaction: High ICH Util × High HL Risk	0.52	(0.30, 0.90)	0.019	3.5
Model performance				
Area Under the Curve (AUC)	0.788	(0.760, 0.816)		
Sensitivity (%)	71.5			
Specificity (%)	74.3			
Correct Classification Rate (%)	73.2			

HL, Health Literacy. Model also adjusted for village bandwidth level and number of chronic conditions. ^**#**^Estimated by proportional decrease in Nagelkerke R^2^ upon variable removal. ^**‡**^High HL Risk’ defined as belonging to the lowest tertile of the predicted health literacy score from a model containing only age, sex, and education.

## 4 Discussion

By using quantile regression framework, combined with mediation analysis and sensitivity analysis, this study reveals that there is a detailed and unbalanced association picture between digital participation in intangible cultural heritage and health literacy among rural residents in China. Four main findings confirmed our original hypothesis: First, in support of Hypothesis H1, the association was positive and significant across the health literacy distribution, but its strength showed a clear gradient, with a large increase in strength for individuals with already high levels of health literacy. Second, consistent with Hypothesis H2, this relationship is mediated by environment and life stage and is significantly stronger in low-bandwidth villages and older adults (≥ 60 years), especially for those at the lower end of the health literacy spectrum. Thirdly, it confirms the hypothesis H3 that digital self-efficacy mediates a considerable part of this association (31.7%), and this mediation path is significantly stronger in older adults and low-bandwidth environments, which provides preliminary empirical support for the ability spillover mechanism. Fourth, the association between high frequency intangible cultural heritage use and reduced risk of inadequate health literacy is moderately robust to potential unmeasured confounders. Taken together, these findings highlight the importance of moving beyond a single, average-effect understanding of digital engagement and steer the discussion toward distribution patterns, underlying mechanisms, and targeting strategies.

### 4.1 Interpreting heterogeneity and mechanism: from association to a testable model

The core methodological contribution of this study is the application of quantile regression, which reveals significant heterogeneity masked by traditional mean-based models. The finding that the association between ICH participation and health literacy amplifies along the health literacy gradient suggests a dynamic process of “skills begetting skills” or cumulative advantages, in part consistent with the “Matthew effect” observed in technology adoption and education (26). Individuals with higher basic health literacy may have better information processing ability, digital manipulation skills or self-efficacy, enabling them to participate in and learn from digital cultural resources more effectively (27). To further contextualize this finding in a rural Chinese setting, evidence from a recent national study of middle-aged and older population people in rural China suggests that digital literacy is positively associated with multidimensional health behaviors, including physical examination, healthy diet, and dietary supplement intake, with heterogeneous effects across age groups and regions (28). This supports our interpretation that digital competencies, once acquired through culturally relevant engagement, can translate into tangible health practices. However, this heterogeneity also reveals a key public health equity consideration: the weakest association for the lowest quantile (Q10) suggests that only universal “access” to digital intangible cultural heritage resources may not be sufficient to substantially improve the health literacy of the most disadvantaged (29). This pattern echoes findings from rural Thailand, where digital exclusion and limited health literacy among older adults are strongly associated with inappropriate self-medication behaviors, highlighting the compounding disadvantages faced by the least digitally literate population (30). Critically, our mediation analysis pushes this reading from speculation to evidence: a significant indirect effect through digital self-efficacy suggests that ICH participation may operate in part by building foundational digital confidence, a transferable capacity, which then facilitates the seeking and understanding of health-related information (31). This path is consistent with the core concept of social cognitive theory, in which mastery experience (here, browsing familiar cultural content) builds self-efficacy, which in turn affects behavioral competence (health information management) (32). In contrast, the relative weakening of the overall association at the lowest quantile, coupled with weaker indirect effects in this group, highlights a key consideration: preexisting capacity deficits may set a minimum threshold for effective skill transfer (33), suggesting that culturally relevant digital content, while potentially beneficial, But more structured, scaffold-supported measures may need to be integrated for the most vulnerable (16).

### 4.2 Contextual and demographic effect modification: a paradox of scarcity and relevance

The stronger correlation observed in the low-bandwidth environment poses a thought-provoking paradox, which is a modification of the infrastructure-centric digital divide theory (34). While more optimal broadband conditions are consistently associated with better digital outcomes, our results suggest that the nature and relevance of digital engagement become critical under resource constraints (35). Lightweight and localized intangible cultural heritage resources can reduce technical and cognitive friction and create a low-threshold and high-relevance digital world entrance. This is in line with the concept of “appropriate technology” in digital development, that is, solutions designed for and operating within constraints can produce meaningful results (36). Our finding that the mediating path through self-efficacy is stronger in low-bandwidth environments further supports the idea that focusing on simple and meaningful content without distracting, complex high-bandwidth applications may lead to a more focused increase in digital confidence (37). This implies that in resource-constrained environments, public health and digital inclusion strategies should perhaps prioritize the development and promotion of such “lightweight” culturally relevant applications as foundational steps (38). Similarly, the more significant association and stronger mediation effect in the older adult group challenges the defect-based technology acceptance model of the older population and is closer to the social emotional choice theory. The theory holds that later in life, motivation shifts to emotionally meaningful goals (39). For this group, the digital content of intangible cultural heritage represents the digitization of life experience, social identity and emotional meaning (40). Thus, engagement may trigger a unique and powerful virtuous cycle: cultural familiarity lowers digital anxiety and increases motivation; successful interactions boost digital self-efficacy; and this enhanced confidence facilitates exploration of other online domains, including health (41). Therefore, our findings advocate the adoption of an asset-based paradigm in digital inclusion for older populations (42), using their rich cultural capital as the main fuel for building digital capabilities, rather than focusing solely on remedying perceived deficiencies.

### 4.3 Theoretical integration and extension: the culturally driven capability spillover model

**Figure 6:**
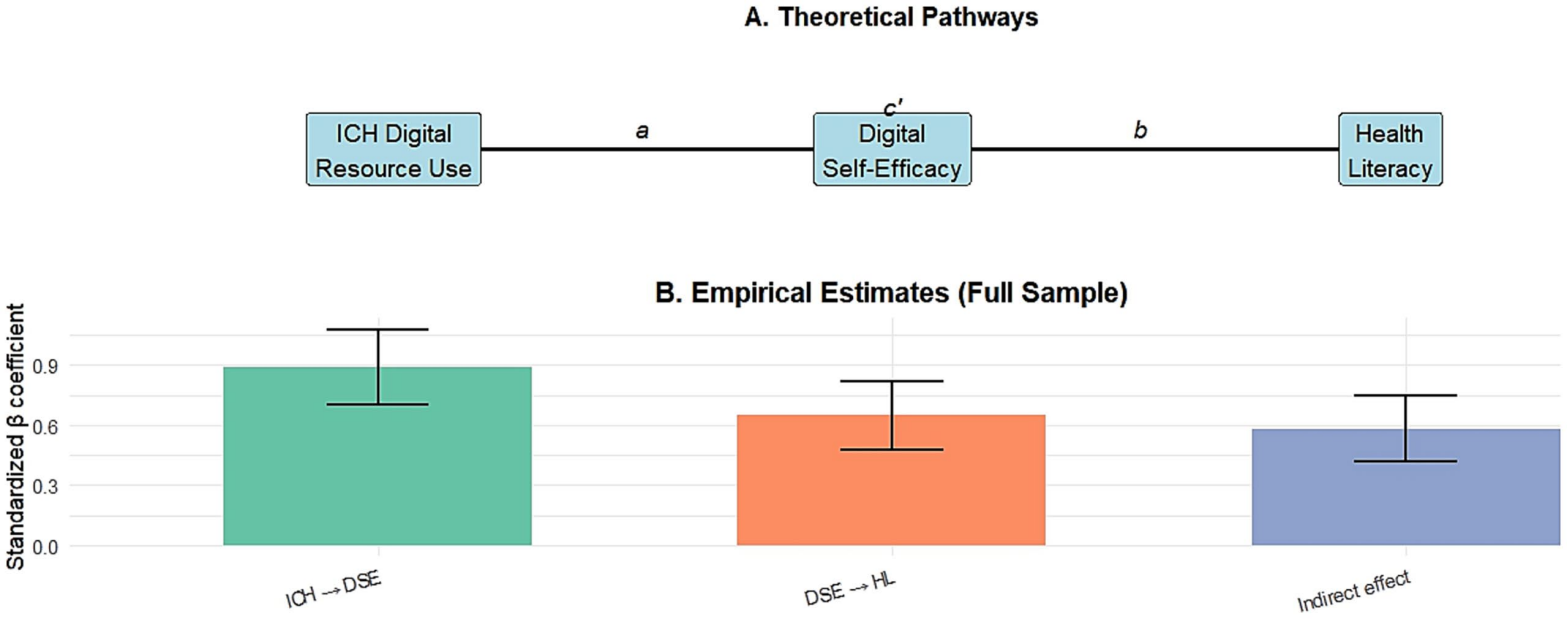
The culturally driven capability spillover model with empirical support. **(A)** A theoretical approach illustrating the hypothesized mediation of digital self-efficacy. The use of ICH digital resources is aimed at improving digital self-efficacy (path a) and, in turn, health literacy (path b). After considering the intermediary factors, path c’ represents the direct impact of intangible cultural heritage use on health literacy. **(B)** Empirical estimates for the full sample (*N =* 1,457) based on mediation analysis. The bars represent the normalized beta coefficients for each pathway, and the error bars represent the 95% confidence intervals derived from bias-corrected self-weight sampling (1,000 replicates). All estimates were adjusted for age, gender, education, household income, village bandwidth level, and number of chronic diseases.

Combining these observations, we propose and provide initial empirical support for “culture-driven capacity spillover” framework (Figure 6). The model argues that participation in digital platforms centered on core cultural value functions (e.g., intangible cultural heritage) in communities undergoing digital transformation serves as a high-motivation, low-threshold practice field for developing generic information capabilities (e.g., browsing interfaces, evaluating content) (43). Intrinsic motivation stemming from cultural relevance sustains engagement, especially in older adults and in settings lacking other digital attractions (2). The competencies developed (here mainly embodied in digital self-efficacy) are essentially transferable information and operational skills (44). These skills, in turn, reduce the perceived and actual barriers to accessing, evaluating, and understanding health information online, enabling functional spillovers from the cultural domain to the health domain (45). The model extends existing theories of the digital divide and health literacy by: (1) identifying a novel, motivation-based entry point for digital skills acquisition (9); (2) emphasizing the transferability of digital self-efficacy as a key mediator (46); and (3) identifying infrastructure and life-stage contexts that amplify this pathway (47). It connects the “access-skills-use” framework of the digital divide with the enabling perspective of health literacy (48) and points out a synergistic path where cultural digitization can serve public health goals (49). Recent empirical studies on the digitization of rural intangible cultural heritage provide evidence of convergence for this model. Zhang et al. (45) demonstrated that heritage-related self-efficacy positively moderates the relationship between cultural identity and responsibility in the context of the digitization of rural intangible cultural heritage, indicating that self-efficacy is a key amplifier of cultural participation effects. This is in direct agreement with our finding that digital self-efficacy mediates the association of intangible cultural heritage health literacy, and that this mediating effect is stronger in older people and in low-bandwidth settings where cultural identity may be more prominent. The convergence of evidence in independent studies supports the plausibility of culture-driven capacity spillover models and suggests their potential generalizability beyond the current research context.

### 4.4 Comparison with prior research and international perspectives

The findings of this study add to a growing body of international literature on how digital engagement, culture, and health outcomes interact in rural and underserved populations. Our results are consistent with a recent study in rural Thailand, where Srisaknok et al. (30) found that digital exclusion significantly affects the health behavior of the older population, and only 14.0% of the older population in rural Thailand in this study were proficient in accessing health information through smartphones. This is consistent with our observation that digital self-efficacy is a key mediator linking cultural engagement and health literacy, suggesting that although cultural content and digital infrastructure are different, similar mechanisms may operate in different rural contexts in Asia. The stronger association we observed among older adults echoes the findings of a nationwide study in China by Li et al. (28), which noted that digital literacy was more strongly associated with health behaviors in middle-aged adults than in older adults, and that these associations varied by region. While Li et al. (5) focused on digital literacy and health behaviors in general, our study advanced the field by identifying a specific pathway through “culturally resonant digital content” and quantifying the mediating role of digital self-efficacy. The convergence of findings across multiple studies highlights the importance of digital literacy as a determinant of health outcomes in older rural populations, and the contribution of our study is to point out that “culturally appropriate digital engagement” may be a potential entry point for intervention. Our findings highlight the unique dynamics of digital engagement in resource-constrained environments, compared to studies in high-income countries where digital infrastructure is more pervasive. Our observation of the “scarcity paradox” - that relatedness is actually stronger in low-bandwidth environments - may be specific to those environments where lightweight, culturally relevant content becomes the primary digital experience. This suggests that digital health equity strategies must be tailored to local infrastructure realities, which is less prominent in studies of areas with full network coverage. Future comparative studies of countries with different levels of digital infrastructure and cultural policies will further clarify how environmental factors shape the link between culture and health.

### 4.5 Implications for research and practice: toward precision public health

Our findings have important implications for both research and practice. For public health research, they advocate that the scope of health literacy interventions should be extended beyond the health sector, and distribution analysis such as quantile regression should be routinely used to reveal inequality (50). Future intervention trials should explicitly examine the causal efficacy of the proposed model — for example, by comparing digital literacy programs based on intangible cultural heritage with standard digital literacy or direct health education programs on digital self-efficacy and health literacy outcomes, with a focus on heterogeneous processing effects (51). For practice and policy, digital inclusion strategies must move beyond a focus on hardware and network connectivity to actively curate, promote, and incentivize people to engage in low-data consuming, culturally resonant content (52). Collaborative initiatives between the public health, cultural heritage and telecommunications sectors are needed (20). Specifically, we propose to launch a “Digital Heritage for Health” pilot project with a precision approach: in low-bandwidth villages, community health workers can help older adults access audio archives of intangible cultural heritage through simple devices. For the general population, this participation alone may trigger spillover effects. However, for individuals identified as high risk (lowest quintile of health literacy), access should be provided in conjunction with optional, low-stress, guided prompts (e.g., “After listening to a story about herbal knowledge, can you find a small suggestion for managing joint pain?”). This hierarchical approach leverages the identified motivational engines while directly addressing the weaker associations found in the most vulnerable populations (53).

### 4.6 Limitations, robustness, and future directions

There are some limitations in this study, and the results should be interpreted with caution to guide future work. First, the cross-sectional design was unable to identify causal inferences; reverse causation (e.g., individuals with higher health literacy or digital self-efficacy actively seek diverse digital content including intangible cultural heritage resources) remains a reasonable alternative explanation for the observed associations. However, our E-value analysis suggests that the observed associations are not easily explained by moderate unmeasured confounding, and that the proposed temporal logic of the mediating relationship (participation in building self-efficacy and, in turn, promoting health literacy) is theoretically sound. Future longitudinal studies, which must measure these variables at multiple points in time, are essential to clarify the direction. Second, while we adjusted for key confounders, unmeasured factors such as intrinsic curiosity, personality traits (e.g., openness to experience), quality of social support for the use of technology, or prior non-digital engagement with intangible cultural heritage may contribute to residual confounding. Third, there may be social desirability bias or recall bias in relying on self-report measures (although verified). Future research must prioritize longitudinal panel studies or intervention studies to examine the causal direction. Promoting experimental trials using adaptive intangible cultural heritage platforms, with random allocation at the individual or village level, is a logical next step. Mixed methods research is critical to understanding the qualitative experiences (emotions, social interactions, and moments of discovery) that underpin quantitative associations. Further research should also explore which features of digital cultural resources (e.g., narrative form, degree of interaction, social sharing function) are most effective in building a sense of digital self-efficacy, and for whom. Finally, research must address the ethical imperatives suggested by our quantile findings: how to design equity-friendly interventions that not only exploit spillover effects but also actively compensate for the weaker associations observed at the bottom end of the health literacy distribution, perhaps through a mentor model that combines digital and artificial, or a co-designed learning scaffold.

## 5 Conclusion

This study shows that active participation in the digital protection of intangible cultural heritage is associated with a higher level of health literacy among rural residents, and brings three key deepening to our understanding of this phenomenon. First, the strength of the association varied significantly across levels of health literacy, increasing from the lowest to the highest subgroups, revealing a pattern of “cumulative advantage” from which those with higher levels of health literacy benefited more. Second, this association is more pronounced in resource-constrained environments (that is, low-bandwidth villages) and among the older population (60 years and older), who are often considered to be digitally disadvantaged. Third, digital self-efficacy mediated about one-third of this, and the mediating effect was also significantly stronger in those subgroups where the overall association was most pronounced.

On the theoretical side, this study makes two innovative contributions. We propose a “culture-driven capability spillover” model and test it empirically. The model argues that engaging in culturally resonant digital content can serve as a high-motivation, low-threshold field of practice to help people develop digital capabilities that can be transferred and applied, which will subsequently enhance their health literacy. This model complements the existing theoretical framework of digital divide and health literacy by identifying a new entry point for the acquisition of digital skills based on motivation and clarifying the mechanisms that amplify this path (i.e., digital self-efficacy) and environmental factors (e.g., infrastructure constraints, life stages).

At the practical level, our findings suggest that we should shift from digital inclusion strategies based on “making up for shortcomings” to approaches based on “leveraging strengths,” that is, making full use of the existing cultural capital of the community. Interventions should not only focus on providing hardware devices or direct health education, but also prioritize the development of lightweight, culturally appropriate digital content, combined with targeted support for the most vulnerable groups. Our proposed tiered strategy, which provides universal access to cultural content for all while providing guided help to high-risk groups, is a precise public health strategy that can effectively address the weaker associations observed at the bottom of the health literacy distribution.

These findings highlight the potential for integrated strategies that incorporate cultural roots to simultaneously advance digital inclusion, cultural sustainability, and public health in marginalized communities. They also illustrate the need for interventions that are not only culturally resonant, but also precisely targeted, to ensure that the dividends of the digital age do not bypass those most in need of them.

## Data Availability

The raw data supporting the conclusions of this article will be made available by the authors, without undue reservation.
