# Supplementary figures and images for: Quantile regression analysis of the heterogeneous association between digital cultural engagement and health literacy: evidence from rural China
Source: Front Public Health. 2026 Apr 9;14:1802126. doi: 10.3389/fpubh.2026.1802126 (PMC13102822; doi:10.3389/fpubh.2026.1802126)

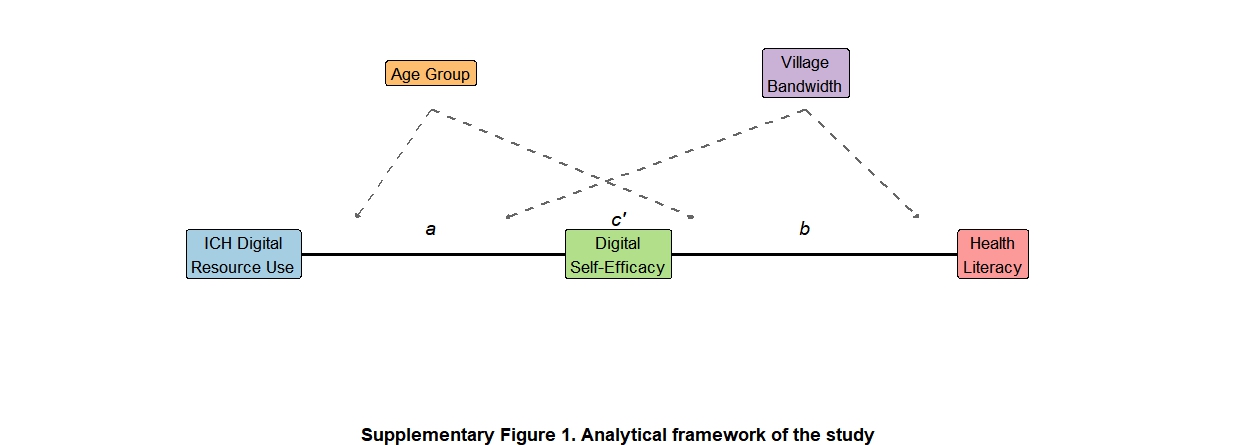

Supplement: Supplementary file 1 [file Image_1.JPEG]
